# *Beagle*: a near-edge X-ray absorption fine-structure spectroscopy data processing solution for beamline experiments at Pohang Accelerator Laboratory

**DOI:** 10.1107/S1600577523008755

**Published:** 2024-01-01

**Authors:** Jae Yeon Park, Minwoong Lee, Seong-Hoon Jeong, Han-Koo Lee

**Affiliations:** a Radiation Fusion Technology Research Division, Advanced Radiation Technology Institute (ARTI)/Korea Atomic Energy Research (KAERI), 29 Geum gu-gil, Jeongeup-si, Jeollabuk-do 56212, Republic of Korea; b Pohang Accelerator Laboratory, Pohang University of Science and Technology (POSTECH), 80 Jigok-ro 127 beon-gil, Nam-gu, Pohang-si, Gyeongsangbuk-do 37673, Republic of Korea; Advanced Photon Source, USA

**Keywords:** NEXAFS spectroscopy, high-throughput analysis, synchrotron radiation beamline, spectral data analysis software

## Abstract

Near-edge X-ray absorption fine-structure (NEXAFS) spectroscopy is a powerful tool for identifying chemical bonding states at synchrotron radiation facilities. Advances in new materials require researchers in both academia and industry to measure tens to hundreds of samples during the available beam time on a synchrotron beamline, which is typically allocated to users. Automated measurement methods, along with analysis software, have been developed for beamlines. Automated measurements facilitate high-throughput experiments and accumulate vast amounts of measured spectral data. The analysis software supports various functions for analyzing the experimental data; however, these analysis methods are complicated, and learning them can be time-consuming. To process large amounts of spectral data, a new analysis software, dedicated to NEXAFS spectroscopy, that is easy to use and can provide results in a short time is desired. Herein, the development of *Beagle* is described, software calculating molecular orientation from NEXAFS spectroscopy data that can report results in a short time comparable with that required to measure one sample at the beamline. It was designed to progress in a single sequence from data loading to the printing of the results with a ‘click of a button’. The functions of the software include recognizing the dataset, correcting the background, normalizing the plot, calculating the electron yield and determining the molecular orientation. The analysis results can be saved as  files (spectral data),  files (graphic images) and *Origin* files (spectral data and graphic images).

## Abstract 2

A new software, *Beagle*, has been developed to analyze NEXAFS spectroscopy at PAL. It calculates molecular orientation for high-throughput experiments, handling more than 100 samples per day.

## 1. Introduction

Near-edge X-ray absorption fine-structure (NEXAFS) spectroscopy is used at synchrotron radiation facilities to reveal the chemical state of research materials, fabricated for both academia and industry (Ade, 1994 ▸; Lee *et al.*, 2008 ▸; Luzio *et al.*, 2014 ▸; Saikubo *et al.*, 2008 ▸; Stöhr, 1991 ▸; Szumilo *et al.*, 2014 ▸; Williams *et al.*, 1980 ▸). In the past, research groups in academia used NEXAFS to analyze small numbers of samples. With the development of new material technologies, especially those related to nanometre-sized and energy storage materials, industry requires large numbers of material samples to be analyzed in a short period (Braun *et al.*, 2007 ▸; Lehmann *et al.*, 2005 ▸; Wen & Jervis, 2022 ▸). Spectral beamlines have evolved for automated measurements and can now measure several samples per day. Automated measurements generate exponentially large amounts of spectral data. For instance, in a NEXAFS experiment, two spectra are created for one sample through total electron yield (TEY) and partial electron yield (PEY) measurements, and a total of eight spectra are generated through four angle-dependent experiments. Thus, in a beamline that measures more than approximately 40 samples per day on average, more than 320 spectra are created (Floreano *et al.*, 1999 ▸; Zheng *et al.*, 2014 ▸). Therefore, beamline users must utilize spectroscopic analysis software that can handle such large amounts of data.

Computer software for analyzing spectroscopic data has been developed with various functions over the last two decades (Newville *et al.*, 1993 ▸; Zabinsky *et al.*, 1995 ▸). The evolution of data analysis software extends from spectral to microscopic analysis, with a range of capabilities for analyzing one-, two- and three-dimensional data sets. *ATHENA* was developed for X-ray absorption spectroscopy (XAS) data processing with background subtraction and plotting. Another tool, *ARTEMIS*, provides extended X-ray absorption fine-structure (EXAFS) data analysis with Fourier transform and plot fitting (Ravel & Newville, 2005 ▸). The *TXM-Wizard* XANES software package has been developed for hard X-ray transmission microscopy data analysis (Liu *et al.*, 2012 ▸), while *MANTiS* supports spectromicroscopy analysis up to the soft X-ray region, including principle component analysis, cluster analysis and matrix approximation analysis (Lerotic *et al.*, 2014 ▸). *Quick as NEXAFS Tool* (*QNAT*), created at the Australian Synchrotron, loads and analyzes NEXAFS spectroscopy data from soft X-ray beamlines (Gann *et al.*, 2016 ▸). These tools report the analysis results as graphs or tables in a document, which can be visualized using specialized plotting software, such as *Origin* (OriginPro, 2022 ▸), *LabPlot* (LabPlot, 2023 ▸) and *MATLAB* (MathWorks, 2022 ▸). These software packages support beamline users and spectroscopy researchers, through various functions, in analyzing and visualizing the experimental data. However, as diverse functions are added to these software, they may accumulate superfluous functionality for a specific experiment. These software tools also provide macro functions to execute a series of commands for repetitive tasks.

Herein, we report the development a data analysis software, *Beagle*, optimized for NEXAFS spectroscopy at the soft X-ray beamline at Pohang Accelerator Light (PAL), Pohang, South Korea. This tool was designed to recognize data sets from angle-dependent experiments, arrange measured NEXAFS spectra, correct energy delay, subtract background noise, calculate molecular orientation, and export calculation results into 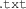 , 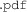 and *Origin* files. The program’s workflow process consists of one continuous sequence from data loading to result exporting. Because this program works at a ‘click of a button’, it is easy to execute, with a fast turn-around time.

## 2. Design of *Beagle* for NEXAFS data analysis

**Figure 1:**
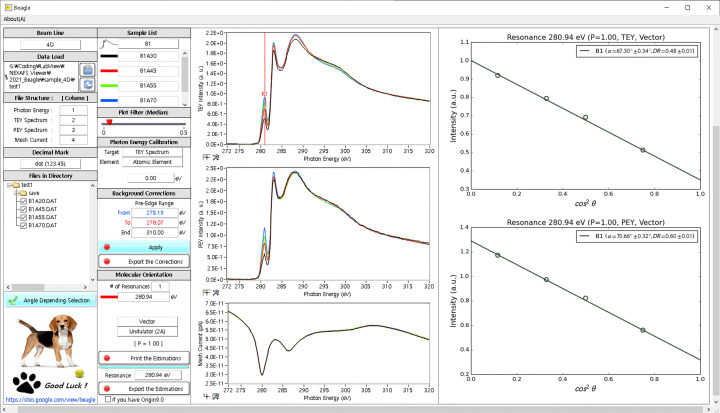
Graphical user interface of *Beagle*.

As shown in Fig. 1 ▸, *Beagle* has been designed to display all functionalities on one screen to the extent possible, instead of forcing the user to switch screens or open new windows. This design approach eliminates the hassle of searching for functions (in other words, no functions are hidden); this allows users to recognize inputs and outputs at a glance. The input and output panels are located in the left two vertical columns and the right two vertical columns, respectively, on the screen. The left-most column presents the input and arrangement of data files and the grouping and listing of files by experimental conditions. The second column from the left shows the plot legends, plot filters, energy calibration, background correction, molecular orientation and output-export options. The user can find more detailed experimental theory, setup and method in the menu bar under software title.

**Figure 2:**
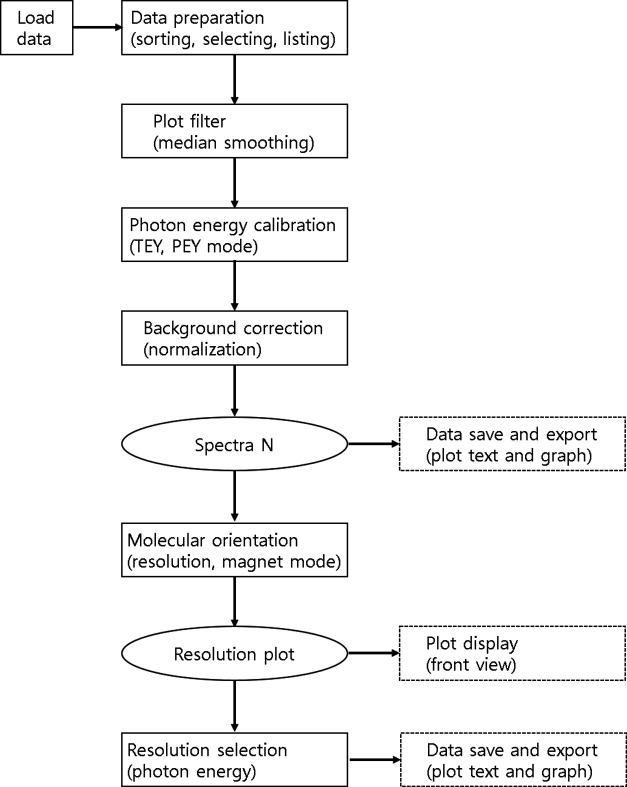
Workflow diagram of *Beagle*.

The structure of the measurement data comprises header information containing the file name, instruments and key measured values in five columns; these include photon energy, TEY, PEY, mesh current and sample actuator position. The column position and column name can be arbitrarily matched to recognize different file structures. This allows *Beagle* to change the conditions of measurement sensors, or load measurement files from other beamlines in a columnar file structure. Typically, hundreds of measurement points are employed, and the measured values are recorded as floating-point numbers. Either a dot (.) or a comma (,) can be selected to represent the decimal point of a floating-point number. The file name of the measurement file includes the experiment name and angle (θ) of the incident photon direction. Note that in the NEXAFS experiment they are used to determine the molecular orientation. Thus, the file-naming convention allows us to recognize the file group. For example, when the experiment is conducted at θ = 30, 45, 55 and 70° with the first experiment named B, the recorded file names would be 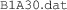 , 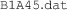 , 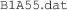 and 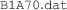 . The character A in the file name represents the angle. The spectral plots and their legends are displayed in the same color as those of the plots and file names. The plot filter in *Beagle* reduces the impulse noise of the plot and renders a smoothing effect using a median filter. This option usually provides only an esthetic modification. The background correction normalizes the X-ray spectrum of the raw data and subtracts the background function. To this end, two energy values in the pre-edge section and one energy value in the end edge are selected by adjusting the vertical line in the plot. The steps starting from the file recognition to the background correction constitute the pre-processing process for molecular orientation calculations. Once all these steps are completed, the clicked buttons become colored and, if those buttons are clicked again, the process state reverts and the button colors disappear. The resonance points depend on the incidence angle, and their line-fitting plot is displayed on the front panel of *Beagle*. This plot displays cos^2^θ and intensity on the *x*- and *y*-axes, respectively, along with the plot legend, file name, tilt angle (α), dichronic ratio (DR), resonance energy, polarization ratio (*P*), mode (TEY or PEY) and magnet type. All the results, displayed on the screen, are saved as 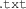 and 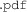 files, that can be printed, or exported as *Origin* files to edit the plot design using *OriginPro*. The 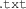 files have a file name indicating experiment name, resonance energy, *P* value and mode type. The data in the 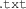 file are the linearly fitted plot with two columns of photon energy and intensity. In the 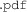 files, the images of the linearly fitted plots shown in Fig. 1 ▸ are saved. The *Origin* files have multiple worksheets and graphs for all data handled in *Beagle*. The complete workflow of *Beagle* is illustrated in Fig. 2 ▸.

### 2.1. NEXAFS analysis with *Beagle*

**Figure 3:**
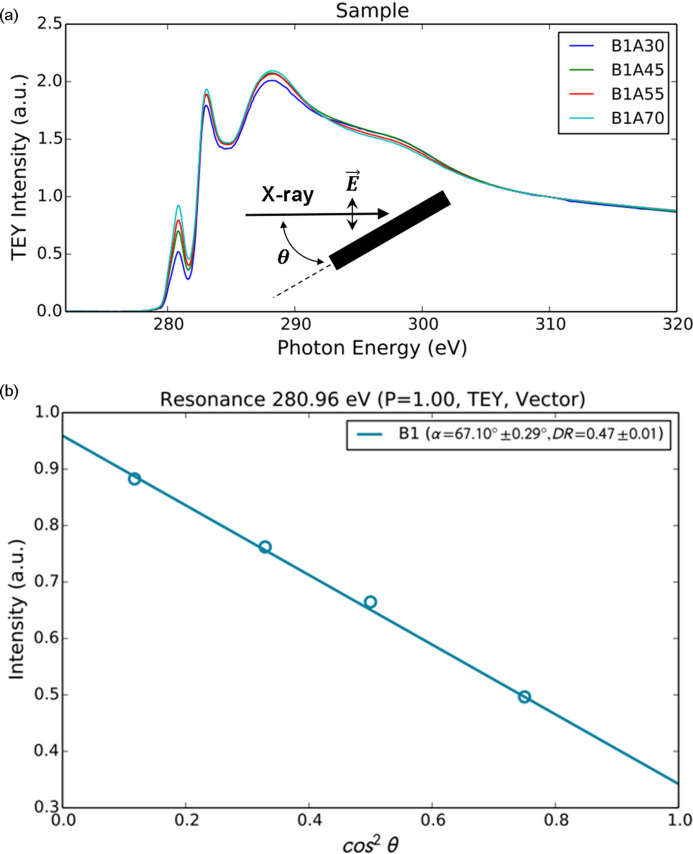
Example results with *Beagle* applied to 10A2 beamline data at PAL. (*a*) Variation of NEXAFS spectra for θ = 30, 45, 55 and 70°. The inset defines θ, which is the angle made by the incident photon beam with the sample. (*b*) Fitting result showing resonance energy, *P*, measurement mode (TEY), molecular orbital (Vector), α and DR.

The NEXAFS spectroscopy facility on the beamline at PAL provides high-resolution power (∼8000), high photon flux (>10^12^ photons s^−1^) and a wide energy range (80–1800 eV). However, to obtain the desired spectroscopic results using *Beagle*, it is necessary to understand the detailed measurement conditions. The X-ray source is generated from a bending magnet or an undulator in a storage ring, and is linearly polarized and chromatized as it passes through the flat mirrors and diffraction gratings in a vacuum (2 × 10^−10^ Torr). The linearly polarized X-rays are used to understand molecular systems, such as low-atomic-number molecules, macromolecules and polymers, which possess directional bonds (Jang *et al.*, 2015 ▸; Lee *et al.*, 2012 ▸; Park *et al.*, 2013 ▸; Stöhr, 1991 ▸). The reference signal *I*_Au_ of the X-rays is measured with an Au mesh, and the sample signal *I*_s_ is measured in the TEY and PEY modes. The NEXAFS spectrum μ(*E*) is calculated based on the ratio of *I*_Au_ and *I*_s_. Figure 3 ▸(*a*) shows sample NEXAFS spectra measured for specific X-ray angle dependence. The absorption edge in the NEXAFS spectra shows the unoccupied orbitals of 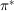 and 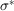 symmetry. The edge intensity of the normalized NEXAFS spectrum depends on the angle (θ) of the incident photon beam polarization vector to the molecular orbital orientation on the sample surface. To characterize the bonding geometry of the vector or plane orbital at resonance energy, the NEXAFS spectra are fitted with the following formulae,

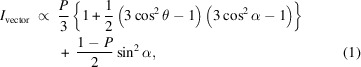




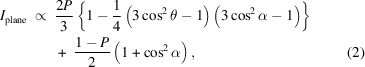

where θ and *P* have been defined earlier. *P* was set to 0.85 using a bending magnet or to 1 using an undulator (Chubar & Elleaume, 1998 ▸; Mathon *et al.*, 2015 ▸). α is the angle between the molecular plane and the substrate plane. The fitting calculation revealed that the intensity of cos^2^θ in the NEXAFS spectra fitting formula varies linearly with intensity, as shown in Fig. 3 ▸(*b*).

### 2.2. High-throughput analysis

**Figure 4:**
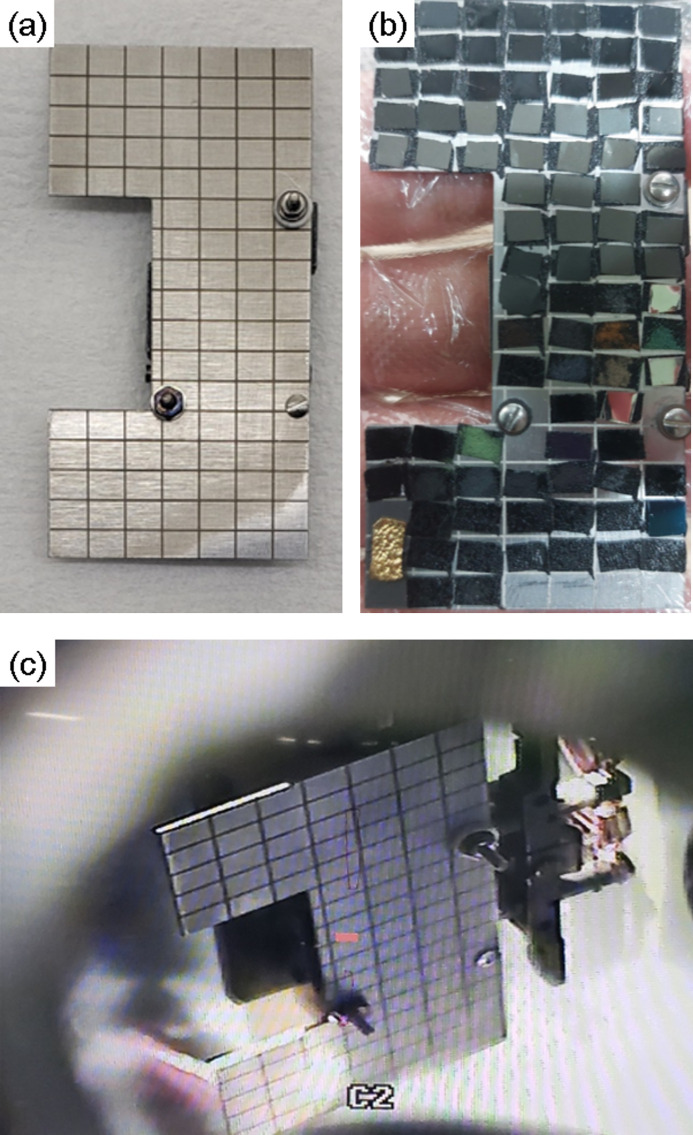
Sample holder for high-throughput experiments. (*a*) 5 mm × 4 mm cell. (*b*) 90 samples. (*c*) Camera display view of the holder installed in a vacuum chamber.

NEXAFS spectral analysis using *Beagle* can allow high-throughput experiments with a sample holder that can hold multiple samples. The measurement using a large sample holder requires only one-time installation of the sample holder in the high-vacuum chamber. The samples are measured with the movement of an actuator in the chamber. Figures 4 ▸(*a*) and 4 ▸(*b*) show a sample holder with cells measuring 5 mm × 4 mm and holding 90 samples. After loading the vacuum chamber with the sample holder constituting the 90 samples in the evening before the beam time, shown in Fig. 4 ▸(*c*), the entire sample measurement can be completed in 15 h or less (approximately two working days), as the measurement time for one sample is less than 10 min. The spectral data analysis of one sample using *Beagle* can be completed in 10 min.

## 3. Further plans for *Beagle*

The development goal of *Beagle* was high-speed analysis for high-throughput experiments. Thus, *Beagle* was designed with a simple graphical user interface for easy usage; it works by a ‘click of a button’. Currently, it is based on the graphical programming environment LabVIEW. The future developmental plans for *Beagle* include the following: (i) incorporating artificial intelligence (AI) for faster and more accurate analysis, (ii) enhancing the capability to load more data structures from other beamlines or spectroscopies, and (iii) porting the tool to an open-source programming environment for compatibility with various computer operating systems. Recently, the scope of AI application was expanded to spectral analysis, which conducts data noise reduction (Kim *et al.*, 2021 ▸), plot trend recognition (Rios *et al.*, 2021 ▸), background correction (Valensise *et al.*, 2020 ▸) and peak position determination (Ghosh *et al.*, 2019 ▸). Using these AI modules or libraries, molecular orientation can be obtained faster and more accurately. Additionally, if the data analysis software is connected to the control software measuring the sample, the analysis results can be derived simultaneously with the measurements. If the calculation results are excessively poor, the AI algorithm may automatically output a caution warning.

*Beagle* can load various data structures, produced from other beamlines or spectrometers. The program part of data loading is modularized, and a *Beagle* user can change the order of the data in the front panel. As the final spectrum data are expressed in terms of energy values and sensing intensities, any data-loading method that is shared or open can be applied to *Beagle*.

*Beagle* was built using the LabVIEW environment in the Windows operating system to facilitate high-speed analysis, rapid development, distribution and quick learning of its operation (Beyon, 2000 ▸; Kalkman, 1995 ▸; Liu *et al.*, 2019 ▸; Yu *et al.*, 2019 ▸). However, the installation size of LabVIEW is relatively large; furthermore, it has poor compatibility with other operating systems. We intend to port *Beagle* to an open-source programming environment so that it can work on different operating systems, and reduce the size of the installation files.

## 4. Conclusion

We have developed *Beagle*, a NEXAFS spectroscopy data analyzer software, optimized for beamlines at synchrotron radiation facilities. The goal of this NEXAFS data analyzer is to quickly obtain analysis results for a large number of samples, while the beamline measurements are being conducted. The analyzer was designed to process spectral data in one sequence on one screen using a ‘click of a button’. The data pre-processing steps include data structure selection, file grouping and impulse noise reduction. The spectral analysis calculates the molecular orientation and angular resonance-line fitting. The results are saved in 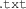 , 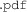 or *Origin* file formats for viewing and editing using conventional documentation software. As this program can handle more than 100 samples per day for high-throughput experiments, it satisfies the requirements of users in both academia and industry, who develop large quantities of new materials. We also plan to port *Beagle* to an open-source programming language that works on all operating systems.
